# Utility and Perceived Value of a Provincial Digital Diagnostic Imaging Repository: Multimethod Study

**DOI:** 10.2196/17220

**Published:** 2020-07-27

**Authors:** Lisa Wickerson, Jamie K Fujioka, Vanessa Kishimoto, Trevor Jamieson, Ben Fine, R Sacha Bhatia, Laura Desveaux

**Affiliations:** 1 University Health Network Toronto, ON Canada; 2 Institute for Health Systems Solutions and Virtual Care Women's College Hospital Toronto, ON Canada; 3 Unity Health Toronto Toronto, ON Canada; 4 Department of Medicine University of Toronto Toronto, ON Canada; 5 Department of Medical Imaging University of Toronto Toronto, ON Canada; 6 Institute of Health Policy, Management and Evaluation University of Toronto Toronto, ON Canada

**Keywords:** diagnostic imaging, eHealth, health care delivery

## Abstract

**Background:**

Timely and comprehensive diagnostic image sharing across institutional and regional boundaries can produce multiple benefits while supporting integrated models of care. In Ontario, Canada, the Diagnostic Imaging Common Service (DICS) was created as a centralized imaging repository to enable the sharing and viewing of diagnostic images and associated reports across hospital-based and community-based clinicians throughout the province.

**Objective:**

The aims of this study were as follows: (1) to explore real-world utilization and perceived clinical value of the DICS following the provision of system-wide access and (2) to identify strategies to optimize the technology platform functionality and encourage adoption.

**Methods:**

This multimethod study included semistructured interviews with physicians and administrative stakeholders and descriptive analysis of the current DICS usage data.

**Results:**

In this study, 41 participants were interviewed, that is, 34 physicians and 7 administrative stakeholders. The following 4 key themes emerged: (1) utilization of the DICS depended on the awareness of the technology and the preferred channels for accessing images, which varied widely, (2) clinical responsibilities and available institutional resources were the drivers of utilization (or lack thereof), (3) centralized image repositories were perceived to offer value at the patient, clinician, and health care system levels, and (4) the enabling factors to realize value included aspects of technology infrastructure (ie, available functionality) alongside policy supports. High-volume DICS usage was not evenly distributed throughout the province.

**Conclusions:**

Suboptimal adoption of the DICS was driven by poor awareness and variations in the clinical workflow. Alignment with physician workflow, policy supports, and investment in key technological features and infrastructure would improve functionality and data comprehensiveness, thereby optimizing health system performance, patient and provider experience, population health, and health care costs.

## Introduction

The ability to electronically share patient-level information across institutional and geographic boundaries can facilitate an integrated and coordinated model of health care delivery among hospital-based and community-based health care professionals by providing timely transfer of relevant and complete information to inform clinical decision making [1]. Within the diagnostic imaging landscape, rapid and comprehensive sharing of imaging data has demonstrated multiple benefits at the patient, clinician, and health care system levels. Patient experience and quality of care is improved by reducing the number of unnecessary tests and radiation exposure [2-5] and by facilitating timely access to specialist consultation and treatment [4]. At the clinician level, timely diagnosis and treatment improves clinical and administrative workflows [2,4,6,7]. Reducing duplicate imaging improves the overall health care system efficiency and associated costs [2-5,8,9]. Technology-enabled models of electronic image sharing include institutional and multi-institutional regional picture archiving and communication systems (PACSs), onsite and offsite vendor neutral archives, cloud-based image transfer, and cross-enterprise document image sharing [10-13]. The technology environment of a health care organization or a clinical practice (ie, technology infrastructure, storage, and resources) influences the platform availability and channels of access for internal and external imaging studies [7]. The import and display of external priors (ie, imaging performed at external institutions) can be time-intensive and lead to clinical inefficiencies, treatment delays, and duplicate testing [2-4]. Physicians may temporarily retrieve and import external imaging data from regional diagnostic imaging repositories (DIRs) directly into their local PACS by using import and display of external priors or foreign exam management [14], whereas community-based health care professionals may rely on accessing a third-party provider portal, image upload from a compact disc or, most commonly, a fax of an image report. Multiple channels of access to diagnostic imaging can lead to variability in clinical and administrative workflow.

In order to alleviate the logistical and administrative burden of transferring imaging data across multiple systems, several jurisdictions have implemented centralized imaging repositories through health information exchanges [13,15]. For example, Scotland has adopted a unified nationwide approach with a single supplier PACS and a central data archive for long-term data storage and sharing [16]. In jurisdictions with fragmented PACSs and electronic medical record systems such as the United Kingdom and Estonia, blockchain technology has been introduced to increase interoperability and decentralize data for easy access and exchange [17]. Blockchain systems have been shown to improve interoperability and reduce administrative costs involved in transporting data, without compromising the security [17]. Within Ontario, Canada, a centralized imaging repository was created by eHealth Ontario, an agency affiliated with the Ontario Ministry of Health, to enable and support real-time sharing and viewing of diagnostic images and reports, which was the focus of this study.

The objective of this clinically focused evaluation was to explore physician engagement and the perceived value following system-wide access to a centralized DIR in Ontario, Canada (the Diagnostic Imaging Common Service [DICS]). This evaluation was completed in partnership with the Ontario Ministry of Health to directly inform future strategies to optimize the technology platform and increase adoption and meaningful use.

## Methods

### Study Design

This multimethod study included one-on-one semistructured interviews that explored the utilization and perceived value of the DICS from the perspective of the physicians across a wide range of specialties and geographic areas. Usage data relating to access of the repository was also examined to understand the utilization of the centralized repository and the practice characteristics of the users who accessed large volumes of data. Ethics approval was obtained from the research ethics board of Women’s College Hospital (REB# 2018-0177-E).

### Study Setting

Medically necessary hospital and physician services (including diagnostic imaging) is publicly funded in Ontario, Canada according to the Canada Health Act [18]. Ontario has a population of over 14 million, which represents 38.6% of the Canadian population in 2018 [19]. There are over 36,000 physicians in active practice in Ontario, including 1000 radiologists and 13,500 primary care physicians [20-22]. In 2012-2013, more than 21 million diagnostic imaging procedures were performed in Ontario, 60% of which were performed in hospitals and the remainder in stand-alone independent health facilities [23]. eHealth Ontario, an agency affiliated with the Ontario Ministry of Health, created the DICS to enable and support real-time sharing and viewing of diagnostic images and reports.

The DICS provides a single front-end web-based viewer that, on the back end, consolidates access to imaging procedures stored across multiple DIRs, thereby providing long-term storage for hospitals and contributing independent health facilities within a specified geography-based catchment area [24]. The DICS is a federated repository that uses a cross-enterprise document sharing to facilitate registration, distribution, and access to images across health care organizations. When a health care practitioner initiates an incoming query to access an image or a report (via a web-based viewer), the cross-enterprise document sharing integration profile sources the relevant data from the DIRs. This enables the health care practitioners to view images and reports across the entire province (ie, outside of their traditional institutional and regional boundaries). To ease the access for these health care practitioners, the DICS was embedded into 3 pre-existing clinical viewing portals as of August 2018. These portals provide the heath care practitioners access to a range of patient-level information, including diagnostic imaging reports, dispensed medications, laboratory results, hospital visits, and home and community care information (ie, referral details, risk assessments, and care plans). The DICS initiative provides the additional ability to view images in a web-based viewer on top of the baseline ability to view image reports. As of January 2019, there were over 35 million images and over 47 million reports available on the DICS. Of these, <0.001% of the images and <0.002% of the reports were accessed during the month of January. While there is some variation based on the clinical viewing portal used, it takes the DICS system an average of 0.49 seconds to respond to a query (range, 0.01-40.85 seconds). This response time is distinct from the time it takes the image to load, which is known to be variable.

### Recruitment and Data Collection

Physicians were recruited using a combination of convenience and purposive sampling to achieve a diverse sample of participants that reflect the breadth of the current use and the future potential of the DICS. The research team, representatives of the Ontario College of Family Physicians, and working group members of the clinical viewing portals were asked to refer contacts who could provide relevant insight. A snowball recruitment strategy was employed, wherein interview participants were asked to refer colleagues who may have relevant insights related to the DICS platform or access to imaging. Purposive data-driven recruitment was also used by asking eHealth Ontario to send out recruitment emails to high-volume users of the DICS based on the viewing statistics. The recruitment expanded to include administrative stakeholders (ie, those with experience in hospital PACS administration, regional DIRs, and independent health facilities) in response to emerging themes around multiple channels of accessing diagnostic imaging and role of the clinical viewing portals in order to fully understand access and engagement. The practice characteristics (ie, geographic location, professional role, and specialty) of high-volume users accessing the DICS during the study timeframe were obtained from eHealth Ontario’s usage statistics.

### Data Analysis

All interviews were audio recorded and professionally transcribed verbatim. Two researchers (LW and JF) independently and inductively coded 3 transcripts to develop a coding framework using NVivo (QSR International), which was applied to the remaining transcripts. Emerging insights were coded inductively and added to the codebook, as necessary. An inductive thematic analysis was applied to identify prominent and recurring themes at regular intervals. Codes were reviewed by 3 members of the research team (ie, LW, JF, VK) who then began the process of thematic mapping to understand the relationships and to generate preliminary themes and subthemes. Refinements and specifications of the thematic categories, subcategories, and relationships between the themes were discerned based on in-depth discussion and negotiated consensus with the fourth member of the research team (LD). Descriptive statistics was performed on usage statistics.

## Results

Between February 22, 2019 and June 30, 2019, 41 participants were interviewed, that is, 34 physicians and 7 administrative stakeholders. The physicians represented a cross-section of the practice areas and a broad range of specialties (Table 1). The administrative stakeholders were managers or directors working in independent health facilities, hospitals, or the regional DIRs who were involved in the implementation or oversight of diagnostic images.

1. Variable utilization is driven by awareness and access preferences: A lack of awareness of prior imaging studies alongside multiple channels available to access internal and external diagnostic imaging and reports (ie, local viewers, regional DIRs, and the DICS through clinical viewing portals) leads to inconsistent and suboptimal engagement with the DICS.
2. Clinical roles and institutional resources inform utilization practices: The functionality of the clinical viewing portals connecting to a centralized DIR needs to fully support the intricacies of the clinical workflow requirements of multiple users, including radiologists, specialists, and primary health care professionals. Radiologists require high-resolution capabilities for images with a link to transcription software to interpret and produce a diagnostic image report, whereas specialists (eg, oncologists, surgeons) use images to plan a surgical approach and monitor change over time in order to plan medical treatments and evaluate responses. When image fidelity, speed of upload, measurement tools, and viewing features for comparative studies (ie, side-by-side viewing) in clinical viewing portals were inferior to local PACS, engagement with the DICS was low. In primary health care, clinicians need efficient and comprehensive access to full reports (rather than the images themselves) to inform their clinical decision making and navigate the patient thorough the health care system. Incomplete or delayed access to reports resulted in decreased utilization. The institutional and technological ecosystem influences how external diagnostic imaging is utilized. While integrating regional DIRs with a local PACS through foreign exam management is a common modality for image viewing, small hospitals and community services may have less technological resources and updated software to support this.
3. Centralized diagnostic imaging was perceived to offer value at the patient, clinician, and health system levels: A centralized DIR with efficient image access was perceived to increase patient satisfaction and safety (ie, reduced radiation exposure, timely diagnosis, and timely treatment), improve the clinical and administrative workflows and communication of the health care professionals, and optimize the health care organizational efficiency by reducing unnecessary repeat imaging and subsequently reducing the health care costs and wait times.
4. Enabling factors to realize value include technology infrastructure and policy supports: High-value technology infrastructure for radiologists and specialists include automatic integration and downloading of images into local systems to enable a comprehensive view of the imaging history, alongside specific functionality of the DICS to support this. Policy supports and infrastructure to promote interoperability between systems (ie, standardization and regulation) and to reduce the image contribution gaps from community-based diagnostic imaging services (ie, independent health facilities) and specific clinical specialties outside of traditional radiology and the scope of DIRs (ie, cardiac imaging) would significantly increase the value and utilization of the centralized repository.

Over 570,000 reports and 135,000 images were viewed between September 2018 and April 2019 across the 3 clinical viewing portals. In February 2019, there were 11,070 users who accessed the DICS at least once during the month. The majority of the high-volume users (566/658, 86%), defined by the research team as viewing >15 images or reports in that month, included health care professionals who were located primarily in the Greater Toronto Area and Southeast Ontario region. This distribution mirrored the demographic profile of the interviewed participants (Table 1).

Four key themes emerged (Table 2), which described physician experiences with accessing images and engaging with the DICS and their overall perceptions of value.

**Table 1:** Participant characteristics (N=41).

Participant characteristics		Physicians (n=34), n (%)	Administrative stakeholders (n=7), n (%)
**Professional specialty**			
	Primary care	7 (20)	N/A^a^
	Radiology	6 (17)	N/A
	Oncology	7 (20)	N/A
	Thoracic surgery	3 (9)	N/A
	Emergency medicine	2 (6)	N/A
	Orthopedic surgery	2 (6)	N/A
	General medicine	2 (6)	N/A
	Respirology	1 (3)	N/A
	Cardiology	1 (3)	N/A
	Urology	1 (3)	N/A
	Gastroenterology	1 (3)	N/A
	Neurosurgery	1 (3)	N/A
**Geographic region**			
	Greater Toronto Area and Southeast Ontario	29 (85)	N/A
	Southwest Ontario	3 (9)	N/A
	Northern and Eastern Ontario	2 (6)	N/A
**Health care setting**			
	Hospital	30 (88)	N/A
	Community	4 (12)	N/A
**Years of clinical practice**			
	0-5	7 (20.5)	N/A
	6-10	7 (20.5)	N/A
	>10	18 (53)	N/A
	Unavailable	2 (6)	N/A
**Role**			
	Administrator for diagnostic imaging repository	N/A	5 (72)
	PACS^b^ administrator	N/A	1 (14)
	Imaging director of an independent health facility	N/A	1 (14)
**Geographic region**			
	Greater Toronto Area and Southeast Ontario	N/A	3 (43)
	Southwest Ontario	N/A	1 (14)
	Northern and Eastern Ontario	N/A	3 (43)

^a^N/A: not applicable.

^b^PACS: picture archiving and communication system.

**Table 2:** Themes describing physician experiences and their illustrative quotes.

Theme	Illustrative quote
Variable utilization is driven by awareness and access preferences	*When I read conceptual imaging, it is like 20, 30, 40 cases a day. I do not know whether there was outside imaging done—if there would be a prompt, that would be very helpful, that is one. It is just not practical. I cannot log in to the clinical viewing portal and check whether any outside imaging was done for every single patient.*[Radiologist 1] *It is my strong preference to use our own viewer in the hospital…because if I can access their images through our (regional DIR*^a^*) interface, and then I can transfer those images to our own PACS*^b^*system.* [Urologist 1]
Clinical roles and institutional resources inform utilization practices	*Ultimately all these web-based viewers, no matter how good they are, they cannot be a PACS quality or caliber machine, because my PACS, yeah, sure, I can do other things on it, but really it is a computer dedicated to 1 task and 1 task only, whereas the website has lots of different tasks.* [Radiologist 4] *I think the local PACS has more functionality in terms of the ability to display multiple images from different dates on the same screen and to synchronize the images so that I can make a direct comparison on 1 monitor between a current scan and a remote scan, like an older scan.* [Thoracic Surgeon 2] *I think just, again, the integration into our workflow is really important. Primary care has a very busy, and a very, very chaotic workflow. If things do not fit into that, they often get left behind, even if they are potentially a helpful resource. I am just really thinking about how to access this in a very easy way, where I do not have to retype in the patient’s date of birth, medical record number, name, etc. Ideally, if it is from the electronic health record, it is connected in. I think that is really important.* [Primary Health Care Professional 5] *There are a few vendors that we do not have foreign exam management set up on yet. I know that they have reached out. They would really like to be able to do that…they do not have foreign exam management and I know that they would be a huge, huge user of it. Once they do decide on the vendor that they are going to use, hopefully we will be able to get foreign exam management set up with them, which would be a really big asset.* [DIR Administrator]
Centralized diagnostic imaging was perceived to offer value at the patient, clinician, and health system levels	* I would say that more often than not, it will lead to one or both of the following outcomes. Number one, the patient care is delayed that day and clinics start running late. That is probably minor. It is annoying and it obviously costs patients more parking money, etc, but the more important one was that I think clinicians just go and say, you know what? Mrs. Robinson, I cannot read this fax. We cannot see your computed tomography image. I am going to call the radiologist. You clearly have an urgent problem and we are going to try to urgently book you for a new computed tomography session here within the next week. So then, clinicians start calling radiologists and they are like hey, I have got a patient in clinic, she is here today, it is kind of urgent, she looks like she has got jaundice, I do not know what is going on, I cannot see the image from the outside, can you just try and find a slot….…I think you guys now can see the downstream impact that this does for health care delivery efficiency…it is huge. And that is just 1 patient’s story but imagine 80 clinicians all doing this all at the same time in 1 center, each 1 individual. You can see how imaging departments across the province are going ugh.* [Oncologist 2].
Enabling factors to realize value include technology infrastructure and policy supports	*I guess what I would need is that my PACS facilitator, the informatics team at my hospital, ideally, automatically, without having to do any work, would automatically be able to import those images in Digital Imaging and Communications in Medicine format from this large system into our system and be matched with the patient’s name so that when I open the study, I can see all the prior imaging data.* [Radiologist 2] *The final downside of the clinical viewing portal is that I cannot transfer the images to my own PACS system. That is always handy because then I can have access to them when I am on the network and if there is network downtime, then also it would allow me to use that internal viewer to do all that stuff more expeditiously.* [Urologist 1] *The biggest downside to all these things is the fact that most outside community-based diagnostic imaging services are simply not available on any electronic format. In particular, in the ultrasound imaging performed outside (ie, independent health facilities), we are missing a vast majority of things that would really be sort of important and would prevent further testing. We order a lot of repeat ultrasounds in patients who have just had an ultrasound, which is a significant drain on resources because unfortunately, the report, I mean I really do not care what the image is but the actual report is simply not available in any repository. So that is kind of the biggest disadvantage is that community access is not there.* [Emergency Physician 2] *It is logical, it is obvious, to be able to look at an echocardiogram and an electrocardiogram with a cardiac magnetic resonance image makes complete sense, and it would be used widely. But currently, the systems are firewalled, you cannot view echocardiograms on our system, and you cannot view nuclear medicine heart testing on our system….* [Radiologist 3]

^a^DIR: diagnostic imaging repository.

^b^PACS: picture archiving and communication system.

## Discussion

### Principal Findings

Although a centralized DIR was perceived to offer clinical value by physicians across a wide range of specialties, there was inconsistent and suboptimal engagement. This was driven by several factors, including a lack of awareness, nuances of clinical workflow and professional roles, functionality of the viewing portals, and policies around interoperability with the local viewing systems. A key driver that was identified to increase clinical utility was the addition of data sources from community-based diagnostic imaging services and other medical specialties to expand the comprehensiveness of the repository. The provision of broad, system-wide access to the DICS through clinical viewing portals was not reflective of, or sensitive to, the heterogeneity of the clinical roles, workflows, and diagnostic image requirements of physician specialists. The inability to upload images from the clinical viewing portals into the local PACS leads to limited utility for the physicians who rely primarily on the PACS for their workflow (ie, radiologists and in-hospital specialists) [25]. Rather than providing similar access and functionality to all the physicians, greater value may be realized if optimization of the digital platform is targeted toward high-priority clinical areas, wherein diagnostic imaging is integral to specific professional roles and workflows. The potential priority areas identified from this evaluation included oncology, surgery, and orthopedics, wherein diagnostic images are routinely used to inform a surgical and medical approach, monitor response to treatment, and track progression/resolution over time. Previous research performed in a surgical oncology center in Ontario found that a shared regional DIR decreased repeat imaging and reduced the waiting times for surgical consultation and surgery [4]. An additional specialized image sharing technology is the Emergency Neuro Imaging Transfer System. This system is a centralized web-based image archive distinct from the DICS that is available in select acute care centers in Ontario that provides temporary access to “on demand” neurological, vascular, and cardiac computed tomography images, magnetic resonance images, and ultrasound images for urgent or critical care [26]. To better understand how to optimize and implement the DICS to increase adoption and utilization, targeted clinician consultation is needed to elucidate value propositions in high-value clinical areas alongside robust clinical and administrative workflow mapping, co-design with intended clinician users [27,28], and education to increase the awareness of the centralized repository.

Beyond the functionality of the DICS and the alignment with the clinical and administrative workflows, a major reported barrier to clinical utility and perceived value of the DICS was the lack of data comprehensiveness. This arose from the reality that community-based independent health facilities do not regularly contribute images to regional DIRs, despite performing up to 40% of the radiology procedures in Ontario [23]. In addition, images that were outside of traditional radiology, such as cardiac images, were not within the scope of the original DIRs and thus were not consolidated into the DICS. Further, multiple channels for accessing imaging data often exist (ie, DIRs, local or regional PACS, and the DICS in Ontario), which can lead to image duplication and fragmented data sets. Consolidated access to comprehensive images and reports through a single portal would produce the most value for clinicians and the health system by facilitating timely access and reducing duplicate imaging [29]. However, administrative stakeholders described challenges surrounding system variation in terminology mapping, data formats, and protocols, storage, and sustainment models for ongoing image contribution from community-based facilities.

Successful implementation of diagnostic image sharing platforms is supported by digital formats and standards that ensure interoperability across multiple vendors and establishing image quality and reporting standards to meet the diverse needs of multiple clinical areas [30-32]. Policy supports around incentives and mandatory reporting of any imaging studies receiving public payment would also encourage data contributions from heterogeneous organizations. Such approaches are best supported by a coordinated government strategy such as the Nationwide Interoperability Roadmap in the United States [33], wherein vendors, health care systems, and medical institutions have committed to information technology exchange standards. Consequently, increasing comprehensive and consistent availability of imaging reports across the system is best facilitated through multistakeholder collaborative efforts, which would, in turn, optimize the clinical value of the centralized repository.

### Limitations

This formative study provides insight into the perspectives of diverse health care professionals, including radiologists, physician specialists, and primary care practitioners. As this study was meant to generate hypotheses about the current use and future potential of the DICS, the next step is to conduct a thorough analysis of the quantitative usage data and identify high users of imaging services in Ontario. In collaboration with the Ontario Ministry of Health, we will identify target users and engage in specialty-specific assessments of imaging requirements and workflow patterns to increase the generalizability of the results. The majority of the participants (34/41, 82.9%) were physicians practicing at academically affiliated hospitals and localized to 1 region of the province. Although this was reflective of the demographic data of the DICS users, future work is needed to understand a broad range of the clinician perspectives as current usage is suboptimal, and current user demographic data are not reflective of the optimal target population. In addition, other types of health care professionals who may access the DICS, such as nurses and allied health care professionals, were not interviewed. The administrative stakeholder group was not the primary recruitment target; therefore, further engagement is needed to build upon their preliminary insights and gain a comprehensive understanding of the administrative and policy drivers that influence engagement with the DICS.

### Conclusions

The clinical utility and perceived value of a system-wide, one-size-fits-all approach to a centralized DIR has not been fully realized owing to suboptimal awareness and lack of alignment with end-user workflows. Further engagement with potentially high-value clinical information users (ie, those who access large volumes of diagnostic images and reports) will help in aligning the technology platform with the nuances of different medical specialist end-user workflows and diagnostic imaging needs. In parallel, investment in data comprehensiveness and inclusion of all imaging reports in Ontario in 1 system would enhance the value by strengthening the utility of the available data.

## Abbreviations

DICS: diagnostic imaging common service
DIR: diagnostic imaging repository
PACS: picture archiving and communication system
